# Objective evaluation of tongue diagnosis ability using a tongue diagnosis e-learning/e-assessment system based on a standardized tongue image database

**DOI:** 10.3389/fmedt.2023.1050909

**Published:** 2023-03-13

**Authors:** Makoto Segawa, Norio Iizuka, Hiroyuki Ogihara, Koichiro Tanaka, Hajime Nakae, Koichiro Usuku, Kojiro Yamaguchi, Kentaro Wada, Akihiro Uchizono, Yuji Nakamura, Yoshihiro Nishida, Toshiko Ueda, Atsuko Shiota, Naoko Hasunuma, Kyoko Nakahara, Miwa Hebiguchi, Yoshihiko Hamamoto

**Affiliations:** ^1^ Department of Kampo Medicine, Yamaguchi University Hospital, Ube, Japan; ^2^ Yamaguchi Health Examination Center, Ogori-shimogo, Japan; ^3^ Department of Computer Science and Electronic Engineering, National Institute of Technology, Tokuyama Collage, Shunan, Japan; ^4^ Department of Traditional Medicine, Faculty of Medicine, Toho University, Tokyo, Japan; ^5^ Department of Emergency and Critical Care Medicine, Akita University Graduate School of Medicine, Akita, Japan; ^6^ Sakura Red Cross Hospital, Kumamoto, Japan; ^7^ Outpatient of Dental Chronic Disease, TANAKA Orthodontic Clinic, Medical Corporation HAYANOKAI, Kagoshima, Japan; ^8^ Division of Nephrology and Dialysis, Department of Internal Medicine, Nippon Kokan Fukuyama Hospital, Hiroshima, Japan; ^9^ Sendai Otorhinolaryngology Clinic, Kagoshima, Japan; ^10^ Shobara Red Cross Hospital, Hiroshima, Japan; ^11^ Department of Obstetrics and Gynecology, Faculty of Medicine, Oita University, Oita, Japan; ^12^ Fukuoka Tokushukai Hospital, Fukuoka, Japan; ^13^ Department of Health Sciences, Faculty of Medicine, Kagawa University, Kitagun, Japan; ^14^ Department of Medical Education, Graduate School of Biomedical and Health Sciences, Hiroshima University, Hiroshima, Japan; ^15^ Women's Clinic Lapport, Hiroshima, Japan; ^16^ Sato Hospital, Akita, Japan; ^17^ Division of Electrical, Electronic and Information Engineering, Graduate School of Sciences and Technology for Innovation, Yamaguchi University, Ube, Japan

**Keywords:** tongue diagnosis, e-learning, diagnostic ability, assessment system, test difficulty, intrarater reliability, interrater reliability, standardization

## Abstract

**Background:**

In Kampo medicine, tongue examination is used to diagnose the pathological condition “Sho,” but an objective evaluation method for its diagnostic ability has not been established. We constructed a tongue diagnosis electronic learning and evaluation system based on a standardized tongue image database.

**Purpose:**

This study aims to verify the practicality of this assessment system by evaluating the tongue diagnosis ability of Kampo specialists (KSs), medical professionals, and students.

**Methods:**

In the first study, we analyzed the answer data of 15 KSs in an 80-question tongue diagnosis test that assesses eight aspects of tongue findings and evaluated the (i) test score, (ii) test difficulty and discrimination index, (iii) diagnostic consistency, and (iv) diagnostic match rate between KSs. In the second study, we administered a 20-question common Kampo test and analyzed the answer data of 107 medical professionals and 56 students that assessed the tongue color discrimination ability and evaluated the (v) correct answer rate, (vi) test difficulty, and (vii) factors related to the correct answer rate.

**Result:**

In the first study, the average test score was 62.2 ± 10.7 points. Twenty-eight questions were difficult (correct answer rate, <50%), 34 were moderate (50%–85%), and 18 were easy (≥85%). Regarding intrarater reliability, the average diagnostic match rate of five KSs involved in database construction was 0.66 ± 0.08, and as for interrater reliability, the diagnostic match rate between the 15 KSs was 0.52 (95% confidence interval, 0.38–0.65) for Gwet's agreement coefficient 1, and the degree of the match rate was moderate. In the second study, the difficulty level of questions was moderate, with a correct rate of 81.3% for medical professionals and 82.1% for students. The discrimination index was good for medical professionals (0.35) and poor for students (0.06). Among medical professionals, the correct answer group of this question had a significantly higher total score on the Kampo common test than the incorrect answer group (85.3 ± 8.4 points vs. 75.8 ± 11.8 points, *p* < 0.01).

**Conclusion:**

This system can objectively evaluate tongue diagnosis ability and has high practicality. Utilizing this system can be expected to contribute to improving learners’ tongue diagnosis ability and standardization of tongue diagnosis.

## 1. Introduction

Medical professionals must continue learning throughout their lives and constantly strive to improve their knowledge and abilities. In recent years, the utility of e-learning has been recognized, and it has been used for improving various diagnostic abilities, including endoscopic image (1) and radiological image (2) diagnoses. Moreover, an individualized learner-led online learning system linked to a specialist certification system has been developed and used for lifelong learning (3). In Kampo, traditional Japanese medicine, the importance of lifelong learning has also been recognized, and doctors need to work to maintain and improve their diagnostic abilities, including tongue diagnosis. To enhance the learning effect, personalized education according to the learner's proficiency level and self-evaluation is important, and this may be fulfilled through e-learning.

In Kampo medicine, the therapeutic strategy is decided based on the patient's pathological condition “Sho,” and tongue diagnosis is an examination technique for estimating it. “Sho” is a pathological condition diagnosed by evaluating the patient's subjective and objective symptoms through the basic concepts of Kampo medicine. Kampo medicine doctors consider the physical and mental conditions comprehensively through four examinations, i.e., inspection, listening and smelling, medical interview, and touch manipulation, to determine the final diagnostic pathology, “Sho,” and administer Kampo medication. Tongue diagnoses are included in the inspection and are considered to reflect physical and mental conditions. The doctor evaluates the color, morphology, and movement of the tongue body and the color, morphology, and dryness of the tongue coating and determines their association with an imbalance of Ki (Qi) (vital life force energy), Ketsu (blood), and Sui (body fluid). Kampo diagnosis is divided into four dichotomic categories: Yin/You (yin/yang), Kyo/Jitsu (deficiency/excess), Kan/Netsu (cold/heat), and Hyou/Ri (exterior/interior) (4, 5).

The normal color of the tongue body is defined as light red in Kampo medicine. Compared with normal color, pale, red, deep red, and purple tongue colors indicate deficiency and cold, heat, advanced heat, and blood stasis, respectively. Normal morphology of the tongue body is defined as a tongue without swelling, atrophy, tooth marks, or cracks. Compared with normal morphology, swelling, atrophy, tooth marks, and cracks indicate water retention and Ki (Qi) deficiency, Ki (Qi) deficiency and Ketsu deficiency, Sui retention and/or Ki (Qi) deficiency, and Ketsu deficiency and/or lack of Sui, respectively (4, 5).

Kampo specialists (KSs) incorporate diverse and complex tongue colors and morphological features to identify pathological patterns. Nevertheless, the following are some issues with tongue diagnosis: (i) It is difficult to acquire its techniques and requires long-term clinical training; (ii) it is a subjective evaluation based on the experience of doctors, and its reliability and objectivity are low; and (iii) a standardized evaluation method for diagnostic ability has not yet been established. Therefore, it is necessary to (i) develop an efficient and standardized training method, (ii) promote the objectivization and standardization of tongue diagnosis methods, and (iii) develop an objective evaluation method for tongue diagnosis. Recently, definitions of tongue findings (6, 7) and standard methods for tongue diagnosis (8, 9) have been proposed, and objectivization and standardization are in progress. However, there are few studies on the education or ability evaluation for tongue diagnosis, and the development of training equipment has not progressed.

We constructed a standardized tongue image database based on the diagnosis results of multiple KSs and developed a tongue diagnosis e-learning system and reported its educational usefulness previously (10). This study aimed to verify the practicality of this system as an objective evaluation tool for tongue diagnosis. In the first study, the answer data of the tongue diagnosis test of 15 KSs were analyzed, and the tongue diagnosis ability and test quality [difficulty level and discrimination index (DI)] were evaluated. Moreover, since the reliability of tongue diagnosis by KSs has not yet been verified, the match rate between diagnoses has also been verified. In the second study, we administered a 20-question common Kampo test and analyzed the answer data of 107 medical professionals and 56 students to the question of tongue color identification.

In this study, we first demonstrated the practicality of this evaluation system for tongue diagnosis. Subsequently, we analyzed the diagnostic data of multiple specialists, clarified the degree of reliability of the tongue diagnosis, and considered the factors that caused the tongue diagnosis to vary. Finally, we showed that this evaluation system is an excellent system that aggregates the diagnostic skills of individual specialists and creates collective knowledge and can be used for the standardization of tongue diagnosis.

## 2. Materials and methods

**Figure 1:**
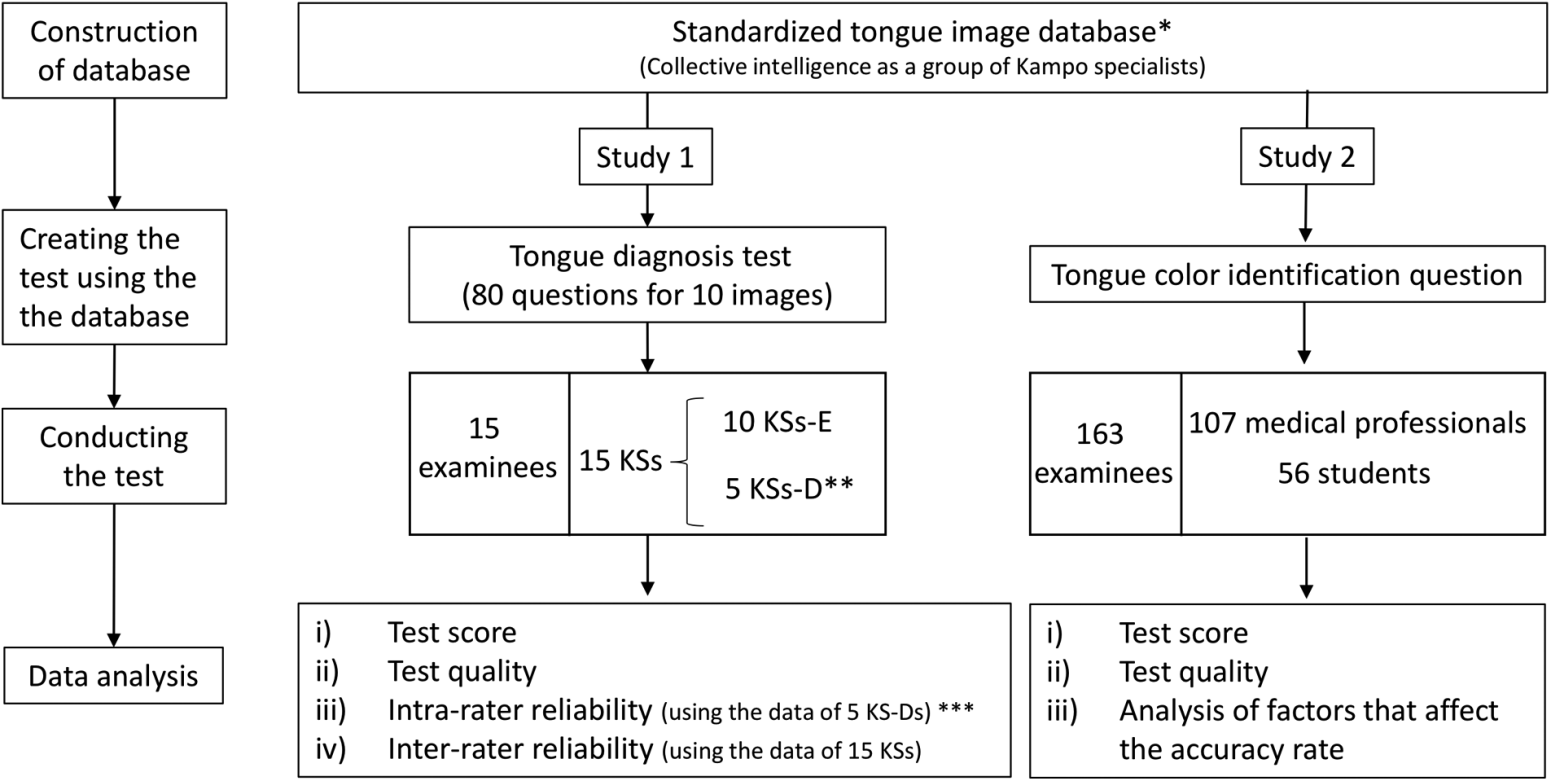
Study design. *Five KS-D participants diagnosed 125 tongue images (first diagnosis). The final diagnosis determined by a majority vote of 5 KS-D participants is linked to the tongue image and constructed database. Details have been reported previously (10). **Five KS-D participants answered the questions of the test (second diagnosis). ***The diagnostic match rate was examined using the data of the first * and second ** diagnoses. KSs, Kampo specialists; KS-D, KS of the database developer group; KS-E, KS of the external evaluator group.

This study was conducted according to the procedure illustrated in Figure 1. This study was evaluated from a scientific and ethical point of view in accordance with the Declaration of Helsinki and the ethical guidelines for medical and health research for humans established by the Ministry of Health, Labor, and Welfare of Japan. This study was also approved by the Ethical Review Committee of the Clinical Research Center of Yamaguchi University Hospital (Research Approval Number: H27-067-3).

### 2.1. Construction of the standardized tongue image database

Details regarding the development of the tongue image acquisition device and the construction of the tongue image database were described in a previous report (10), meaning that they are briefly described below. Five KSs diagnosed the color and morphological characteristics of 125 tongue images from eight viewpoints. The final diagnosis, determined by a majority vote, was linked to the tongue image as highly reliable and objective attribute information. A standardized tongue image database was created using the image and diagnostic information. Data analysis of the diagnosis was performed by a specialist at the Faculty of Engineering.

### 2.2. Construction of the tongue diagnosis e-learning/e-assessment system

**Figure 2:**
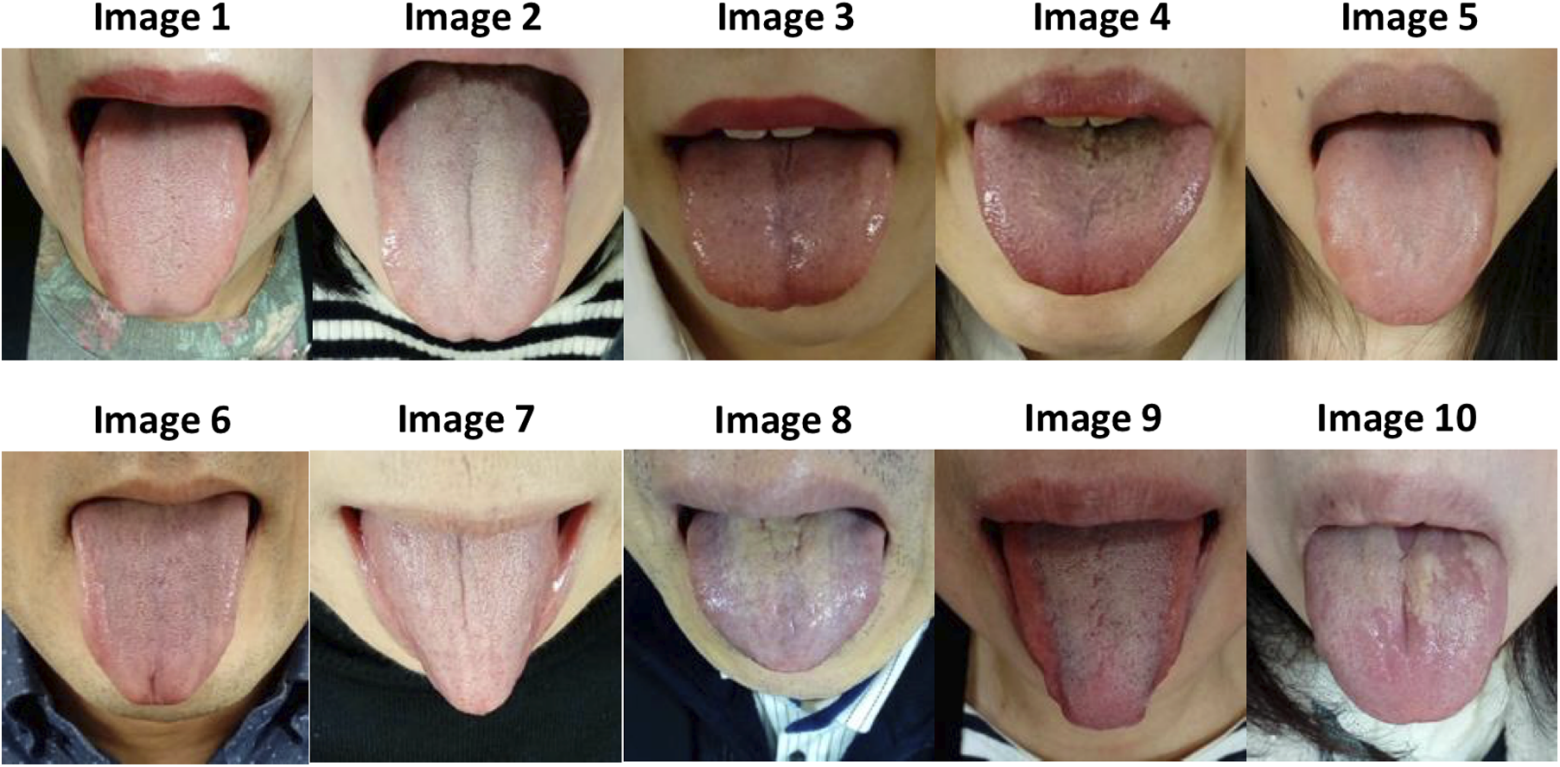
Tongue images of the tongue diagnosis test. (Study 1).

Next, we developed a tongue diagnosis e-learning/e-assessment system that utilizes images from a quality-guaranteed tongue image database (10). This system makes it possible to learn without restrictions on time, place, and equipment and perform self-evaluation of tongue diagnostic ability. The steps are shown below: (1) access an e-learning website; (2) prelearning: confirm the eight standard tongue images displayed on their device before taking the test; (3) tongue diagnosis test: questions about the presented tongue image are shown in Figure 2; (4) send answer data and check grades: immediately after sending the answer data, check the score, correct/incorrect display of all questions, and eight-item radar chart; (5) confirmation of deviation value in the test group (10).

### 2.3. Creating the tongue diagnosis test

#### 2.3.1. Study 1

To evaluate the ability to interpret tongue color, morphology, and condition, an 80-question tongue diagnosis test (Figure 2, Table 1, Supplementary Table S1) was created to diagnose eight viewpoints. Eight questions and answer choices are as follows: (i) Select the size of the tongue in the presented tongue image: normal/swelling/thin/not applicable/unevaluable due to insufficient tongue protruding out of the mouth; (ii) select the color of the tongue body color: pale/light red/red–deep red/purple/not applicable; (iii) select the dryness and wetness of tongue body: normal/dry/wet/not applicable; (iv) select the tooth marks on the edge of the tongue: none/mild/severe/not applicable; (v) select the cracks on the surface of the tongue: none/mild/severe/not applicable; (vi) select the thickness of tongue coating: none/normal/moderate/moderate or higher with peeling/thick/not applicable; (vii) select the color of tongue coating: white/yellowish white–light brown/yellow/dark brown–black/not applicable; and (viii) select the dryness and wetness of tongue coating: normal/dry/wet/not applicable. Abnormalities of Sui (body fluid) cause changes in the color and morphology of the tongue body and tongue coating (11). Therefore, in this study, both the tongue body and tongue coating were evaluated for dryness and wetness of the tongue.

**Table 1:** Answer of the 80-question tongue diagnosis test determined by 15 KSs (Study 1).

	Image 1	Image 2	Image 3	Image 4	Image 5	Image 6	Image 7	Image 8	Image 9	Image 10
Tongue body size	Normal	Normal	Swelling	Swelling	Swelling	Thin	Thin	Normal	Normal	Swelling
Tongue body color	Light red	Pale	Red to deep red	Red–deep red	Light red	Light red or Purple	Pale	Purple	Red–deep red	Light red
Dryness and wetness of tongue body	Normal	Normal	Wet	Normal	Normal	Dry	Normal	Wet	Dry	Wet
Tooth marks on the edge of the tongue	None	None	None	None	Severe	None	None	None	None	None or Mild
Cracks on the surface of the tongue	None	None	Mild	Mild	None	Mild	None	Mild	None	Mild
Thickness of tongue coating	Normal	Normal	None	Moderate	Normal	Normal	Normal	Thick	Moderate	Moderate or higher with peeling
Color of tongue coating	White	White	White	Yellow	White	White	White	Yellow	White	Yellowish white– light brown
Dryness and wetness of tongue coating	Normal	Dry	Wet	Normal	Dry	Dry	Normal	Wet	Dry	Normal or Wet

KSs, Kampo specialists.

The answer was automatically determined by a majority vote of 15 KSs.

Following examination, the average value of the examinee's grade and all examinees’ grades are displayed on the radar chart for each of the eight viewpoints to ensure that they can objectively grasp the level of their diagnostic ability. The clinical significance of each tongue finding has been previously described (10).

#### 2.3.2. Study 2

**Figure 3:**
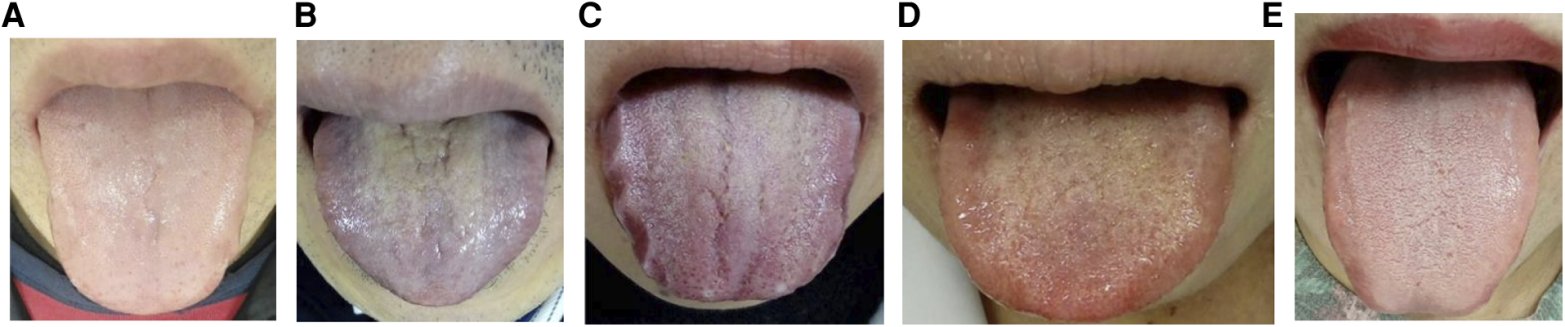
Multiple-choice questions to ask for the normal color tongue (Study 2). Q. Which tongue color is often seen in healthy people? Please select one. The correct answer is (e). The answer distribution of medical professionals was (**A**) *n* = 16 (15%); (**B**) *n* = 0; (**C**) *n* = 0; (**D**) *n* = 4 (3.7%); and (**E**) *n* = 87 (81.3%). The answer distribution of the students was (**A**) *n* = 10 (14.8%); (**B**) *n* = 0; (**C**) *n* = 0; (**D**) *n* = 0; (**E**) *n* = 46 (82.1%).

Multiple-choice questions were created to diagnose “tongues of normal color often seen in healthy people” (Figure 3). The purpose of this question was to evaluate (1) the knowledge of the relationship between tongue color and pathological conditions and (2) the ability to discriminate tongue color. Examinees were to select one of the five types of tongue images [(a) pale; (b) purple; (c) red to deep red; (d) red to deep red; and (e) light red], with option e being the correct answer. Pale or light white indicates blood deficiency, purple indicates blood stasis, and red indicates a heated state. Information on the five types of colors was based on the standardized tongue diagnosis database.

### 2.4. Test implementation and data analysis

#### 2.4.1. Study 1: assessment of tongue diagnosis ability of KSs

##### 2.4.1.1. Test score

We asked 15 KSs to take the tongue diagnosis test (Q1–Q80; Table 1, Supplementary Table S1) and analyzed the answer data. Of these KSs, ten were from the KS-External Evaluator (KS-E) group and five were from the KS-Database Developer (KS-D) group. There were 10 men and 5 women, with ages ranging between 40 and 60 years. All specialists had a wealth of knowledge and experience in Kampo medicine and tongue diagnosis. The test scores, score distributions, and correct answer rates for the eight aspects were calculated. Furthermore, based on the answer results of 15 KSs, the answer was redetermined by a majority vote, and the rate of change in the test answer was calculated. They took an online test using their computers. A standard tongue image (10) was attached to this online test and was available as necessary. Differences in image quality between displays were compensated by using this image as a standard for color and brightness. A color vision test of the 15 KSs was not conducted from the viewpoint of personal information protection.

##### 2.4.1.2. Difficulty level and discrimination index of the test

The difficulty level and identification index of the 80 questions were calculated, and the quality of the test was evaluated. The difficulty level was defined as the percentage of test takers who answered correctly (12). In this study, a correct answer rate of 85% or more was defined as easy, a rate of 50%–85% as moderate, and a rate of <50% as difficult. The DI is an indicator that distinguishes between those with high and low scores (13). The fourfold point correlation coefficient (*φ* coefficient) was calculated as the number of correct and incorrect answers in the top 25% and bottom 25% of grades. In this study, a *φ* coefficient of <0.1 was defined as defective, 0.1–0.3 was defined as fair, and ≥0.3 was defined as good. The *φ* coefficient was calculated using the following formula: $\varphi =(A\times D-B\times C)/\sqrt{\left(A+B)(C+D)(A+C)(B+D\right)}$, where A is the number of correct answerers in the group with the top 25% of the total score, B is the number of wrong answerers in the group with the top 25% of the total score, C is the number of correct answerers in the group with the bottom 25% total score, and D is the number of incorrect answerers in the group whose total score is in the bottom 25%.

##### 2.4.1.3. Intrarater reliability

To evaluate intrarater reliability, the answer data for the tests of the five KSs in the KS-D group were compared with the first diagnosis data in the database construction, and the diagnostic match was calculated. The interval between the first diagnosis in the database construction and the second judgment for tests was approximately 3 months. Moreover, the correlation between the intrarater diagnostic match rate and rater's test score was evaluated.

##### 2.4.1.4. Interrater reliability

To investigate the reliability of tongue diagnosis, the degree of diagnostic agreement among the 15 KSs was evaluated using Gwet's agreement coefficient 1 (AC1) (14, 15), which is a statistical index of interrater reliability.

#### 2.4.2. Study 2: evaluation of the ability to discriminate tongue color of medical professionals and students

An online lecture was conducted according to the contents of the textbook by Kampo (16) at the 71st Annual Meeting of the Japan Society for Oriental Medicine. After the lecture, a common Kampo test, comprising 20 questions related to various fields of Kampo medicine, was conducted. An outline of the data analysis of the 20-question test was reported in a previous report (17). In this study, we focused on one question related to tongue diagnosis used in the Kampo common test and performed an additional analysis. In this study, we analyzed the answers of 107 medical professionals and 56 students of the tongue color identification question (Table 2, Figure 3) included in the abovementioned test and analyzed the test score, correct answer rate, factors related to the correct rate, and quality of the questions, including the difficulty level and DI. The background factors of the correct and incorrect answer groups were compared, and factors related to the correct answer rate were searched.

**Table 2:** Degree of difficulty and discrimination index of the questions (Study 1).

		Degree of difficulty (correct answer rate)		
		Hard (0%–50%)	Moderate (50%–85%)	Easy (85%–100%)
Discrimination index	Good (>0.3)	Q11, Q16, Q30, Q31, Q32, Q36, Q40, Q42, Q43, Q45, Q48, Q62, Q63, Q67, Q72, Q76, Q77	Q8, Q10, Q12, Q15, Q20, Q27, Q35, Q34, Q38, Q39, Q46, Q47, Q52, Q54, Q55, Q56, Q60, Q71, Q79	Q7, Q28, Q44, Q64, Q66
	Fair (0.1–0.3)	Q41, Q53, Q57, Q75	Q13, Q14, Q49, Q51, Q65, Q68, Q70	
	Poor (<0.1)	Q17, Q22, Q23, Q26, Q50, Q69, Q80	Q6, Q9, Q24, Q25, Q29, Q33, Q61, Q74	Q2, Q4, Q21
	Undecidable			Q1, Q3, Q5, Q18, Q19, Q37, Q58, Q59, Q73, Q78

The degree of difficulty is the percentage of test takers who answered the question correctly. Here, the correct answer rate of 85% or more is defined as easy, 50%–85% is defined as moderate, and less than 50% is defined as difficult. The discrimination index is an index that distinguishes between those with high scores and those with low scores. The fourfold point correlation coefficient (φ coefficient) is calculated from the answer results of the top 25% and the bottom 25%. Here, a φ coefficient of less than 0.1 is defined as poor, 0.1–0.3 is defined as fair, and 0.3 or more is defined as good.

### 2.5. Statistical analysis

Student's *t*-test was performed to test the difference in the population mean of the two groups consisting of continuous data (quantitative data). The chi-squared test was performed to test the independence of two variables comprising categorical data (qualitative data). The correlation of bivariate continuous data was evaluated using Pearson's correlation coefficient. A *p* value <0.05 was considered statistically significant. Statistical analyses were performed using PAWS Statistics version 18 (SPSS Inc., IBM, Armonk, NY, United States). The interrater reliability of diagnosis was evaluated using Gwet’s AC1 (14, 15). Gwet's AC1 statistic was calculated using online statistical software AgreeStat360 (AgreeStat Analytics, Gaithersburg, MD, United States). The relative strength of the reliability of Gwet's AC1 coefficient was interpreted using the Landis and Koch criteria (18, 19). A Gwet’ AC1 coefficient <0.00 was considered poor, a coefficient of 0.00–0.20 as slight, a coefficient of 0.21–0.40 as fair, a coefficient of 0.41–0.60 as moderate, a coefficient of 0.61–0.80 as substantial, and a coefficient of 0.80 as almost perfect.

## 3. Results

### 3.1. Study 1

#### 3.1.1. Test score and diagnostic match rate

**Figure 4:**
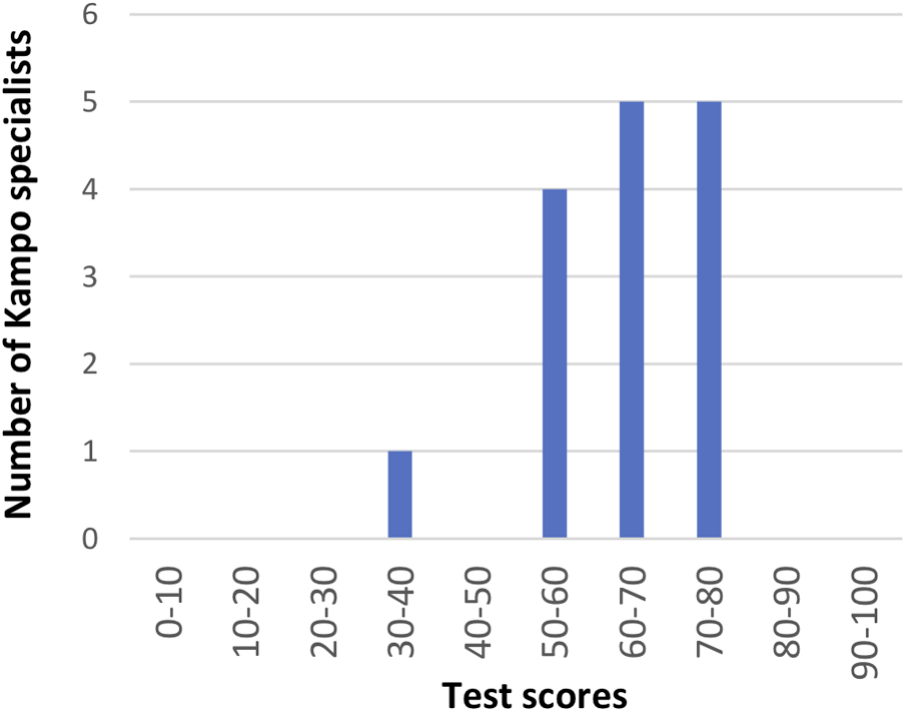
**Results of the tongue diagnosis test (Study 1): Distribution of test scores for 15 KSs.** The average score was 62.2 ± 10.7 points.

**Figure 5:**
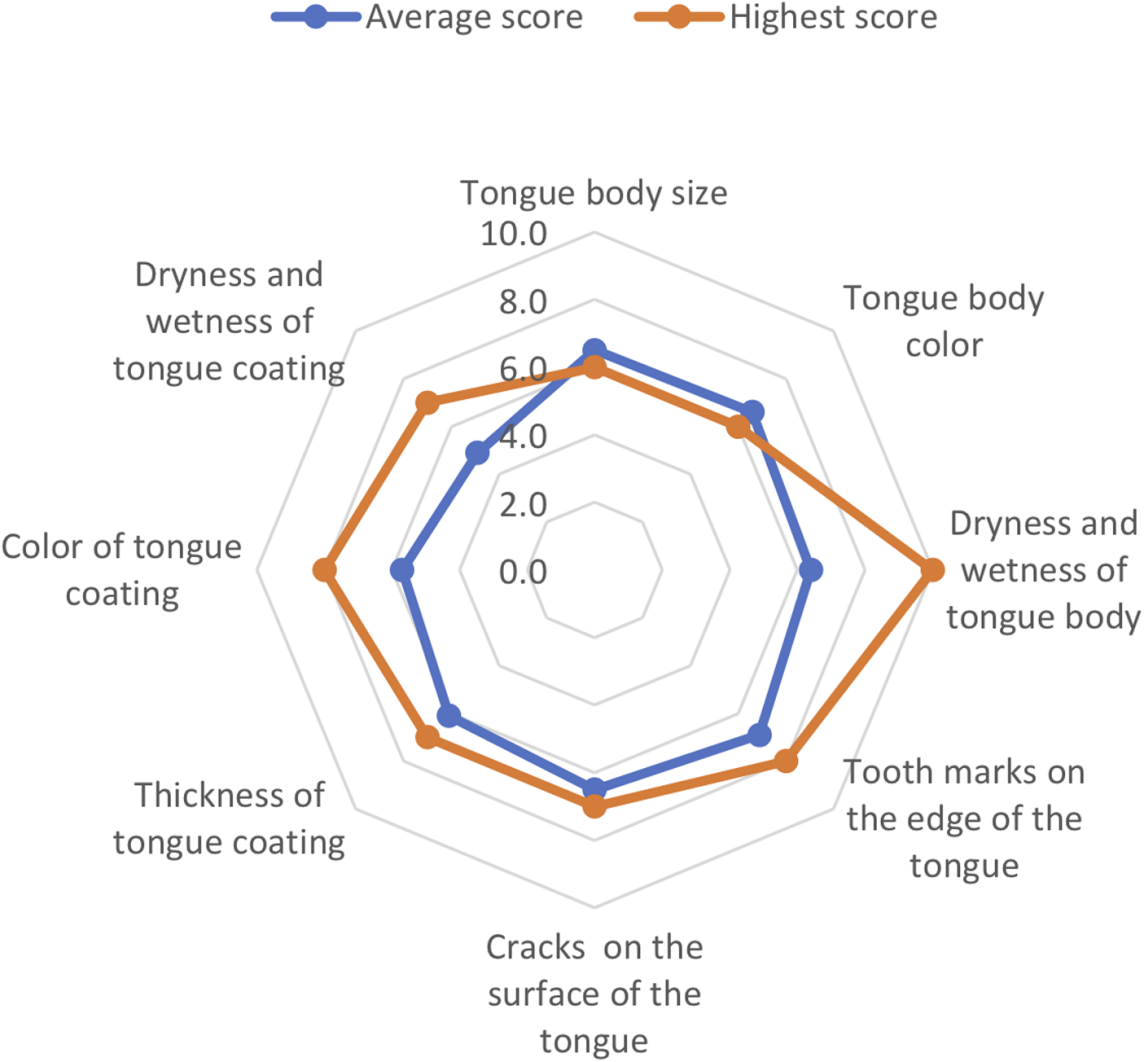
**Results of the tongue diagnosis test (Study 1): the correct answer rate of 8 aspects** Rader chart shows the correct answer rate of 8 aspects. The average number of the correct answer of 10 questions of 15 KSs for each aspect are as follows: 6.5 for tongue size, 6.6 for tongue color, 6.4 for dryness and wetness of the tongue coating, 6.9 for tooth marks, 6.5 for cracks, 6.1 for tongue coating thickness, and 4.9 for dryness and wetness of the tongue body. The data of the highest scorer (KS-E) is also shown. After the test is completed, the test taker's own grades and the average value of all test takers Evaluation of tongue diagnosis ability are displayed on the same chart. Therefore, the test taker can grasp his/her level compared to remaining participants in the group.

The responses of the 15 KSs included are listed in Table 1 and Supplementary Table S1. The distribution of the test scores is shown in Figure 4. If the examinee answered all questions perfectly, the test score was set to 100 points. The average score of the 15 KSs was 62.2 ± 10.7 points. The average scores of the 10 KS-E and 5 KS-D participants were 64.6 ± 9.3 and 57.5 ± 12.9 points, respectively, and there was no statistically significant difference between both groups (*p* = 0.31). The average number of correct answers out of 10 questions from 15 Kampo specialists for each aspect is as follows (Figure 5): 6.5 for tongue size, 6.6 for tongue color, 6.4 for dryness and wetness of the tongue coating, 6.9 for tooth marks, 6.5 for cracks, 6.1 for tongue coating thickness, and 4.9 for dryness and wetness of the tongue body. When the answer was redetermined by a majority vote of the answer results of 15 KSs, the number of questions whose answer was changed was 22 (27.5%) (Supplementary Table S1). According to the judgment of the tongue colors by a majority vote of the 15 KSs, the tongue images were classified, as shown in Figure 7.

**Figure 6:**
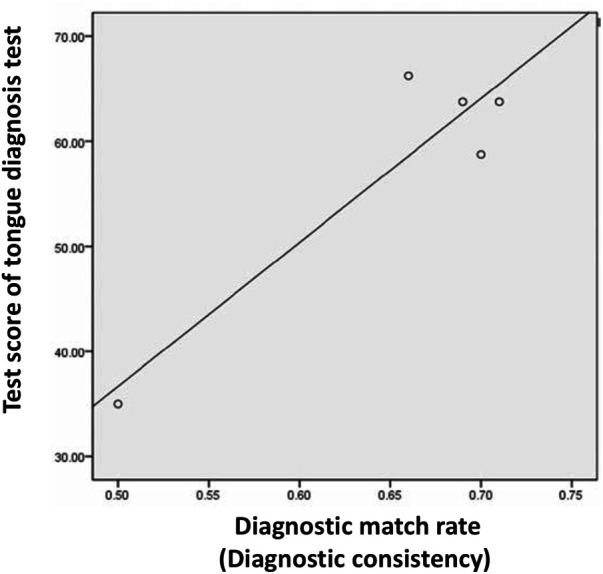
Correlation between diagnostic consistency and test score (Study 1). A high correlation was found between the diagnostic match rate and tongue diagnosis test score (Pearson's correlation coefficient, 0.927; *p* = 0.024).

**Figure 7:**
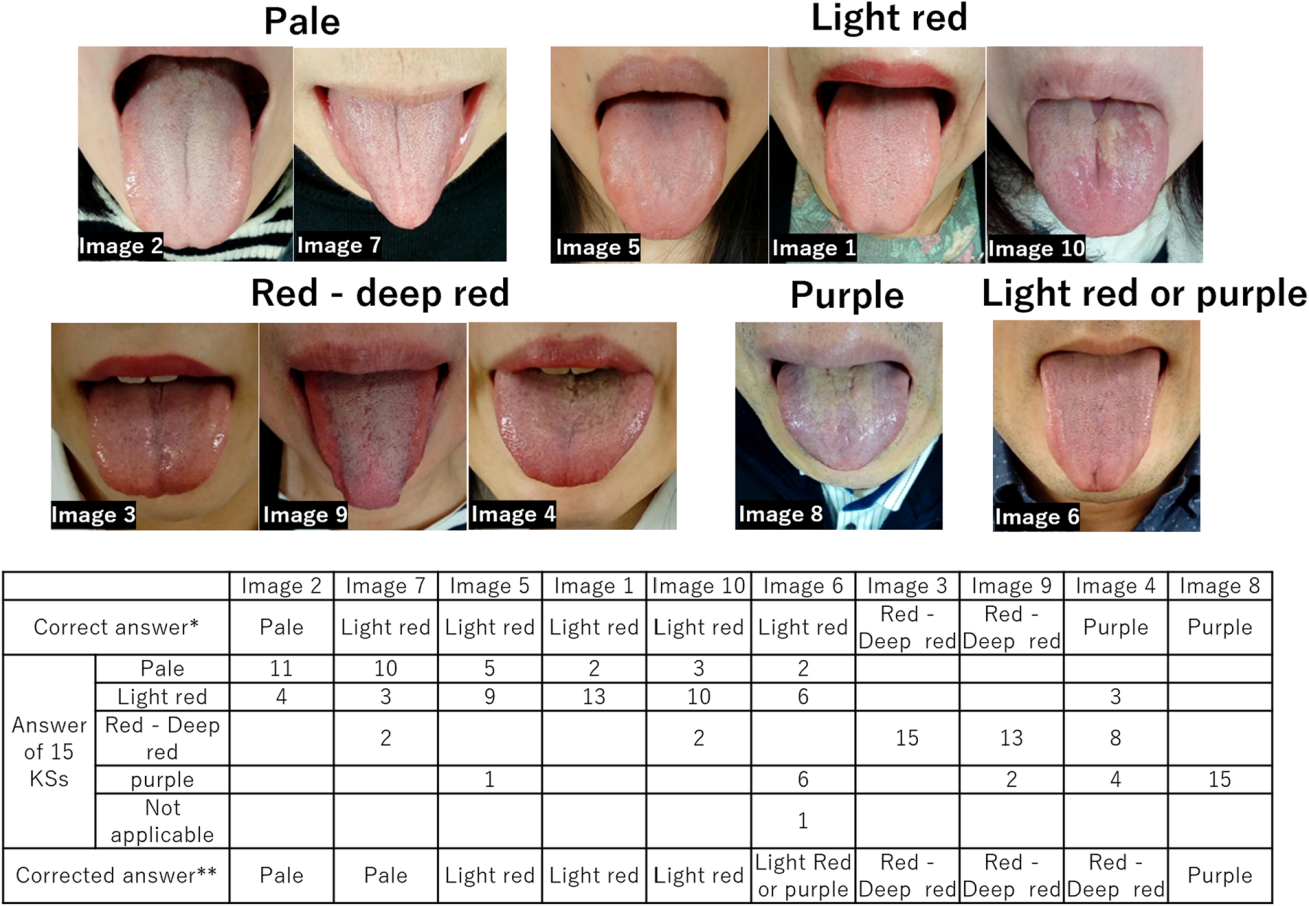
Grouping of tongue color based on a majority vote (Study 1). The correct answers for Q50(Image7), Q42(Image6), and Q26(Image4) were corrected when the decision was made again by a majority vote of 15 KSs. The tongue images classified based on the majority vote are shown above. *Correct answer is based on the information in the tongue image database, which was automatically determined by a majority vote of the diagnostic results of five Kampo specialists (KS-D participants). **Corrected answer is redetermined by a majority vote of the diagnostic results of 15 KSs. KSs, Kampo specialists; KS-D, KS of the database developer group.

#### 3.1.2. Difficulty level and DI of the test

Table 2 lists the difficulty level and DI of the test. Regarding the difficulty level, 28 questions were difficult (correct answer rate: <50%), 34 questions were moderate (correct answer rate: 50%–85%), and 18 questions were easy (correct answer rate: ≥85%). Regarding the DI of the test, 41 questions were good (*φ *> 0.3), 11 questions were fair (0.1 < *φ* < 0.3), 18 questions were bad (*φ* < 0.1), and 10 were undecidable (i.e., the *φ* value could not be calculated as the answer results of the upper 25% and lower 25% of grades were the same).

**Table 3:** Interrater reliability analysis (Study 1).

	AC1 coefficients (95% CI)	Interpretation	Landis and Koch interpretation	
			Range of values	Interpretation
Tooth marks on the edge of the tongue	0.53 (0.36–0.70)	Moderate	(0.8 to 1)	Almost perfect
Tongue body color	0.52 (0.30–0.74)	Moderate	(0.6 to 0.80)	Substantial
Tongue body size	0.47 (0.30–0.64)	Moderate	(0.4 to 0.6)	Moderate
Cracks on the surface of the tongue	0.46 (0.23–0.69)	Moderate	(0.2 to 0.4)	Fair
Thickness of tongue coating	0.45 (0.28–0.62)	Moderate	(0 to 0.2)	Slight
Color of tongue coating	0.40 (0.21–0.58)	Moderate	(−1 to 0)	Poor
Dryness and wetness of tongue body	0.38 (0.14–0.63)	Fair
Dryness and wetness of tongue coating	0.27 (0.08–0.45)	Fair
All images	0.52 (0.38–0.65)	Moderate

Interrater reliability was evaluated by Gwet's agreement coefficient. Gwet's AC1 was calculated using the online statistical software AgreeStat360. Using the Landis–Koch interpretation criteria, the interrater reliability of the dryness of the tongue and the dryness of the tongue coating were interpreted as fair, whereas those of the remaining items were interpreted as moderate.

#### 3.1.3. Intrarater reliability of answers

The diagnostic match rates for the five KS-D participants were 0.51, 0.66, 0.69, 0.70, and 0.71, respectively. The mean rate was 0.66 ± 0.08. The average diagnostic match rate for the eight aspects was 0.83 for tongue size, 0.70 for dryness and wetness of tongue body, 0.67 for dryness and wetness of tongue coating, 0.67 for tooth marks, 0.63 for cracks, 0.53 for tongue body color, 0.53 for tongue coating thickness, and 0.40 for tongue coating color. A high correlation was found between the diagnostic match rate and tongue diagnosis test score (Pearson's correlation coefficient, 0.927; *p* = 0.024) (Figure 6).

#### 3.1.4. Interrater reliability of answers

As shown in Table 3, the AC1 coefficient was 0.53 for tooth marks, 0.52 for tongue body color, 0.47 for tongue body size, 0.46 for cracks, 0.45 for tongue coating thickness, 0.40 for tongue coating color, 0.38 for dryness and wetness of the tongue body, and 0.38 for dryness and wetness of tongue coating, and the ACI coefficient for all images was 0.52. Using the Landis–Koch interpretation criteria, the interrater reliability of two items, dryness and wetness of the tongue body and dryness and wetness of tongue coating, was fair, whereas that of the remainder of the items was moderate.

### 3.2. Study 2

#### 3.2.1. Correct answer rate and factors related to the correct answer rate

Among the medical professionals, the average score of the Kampo common test was 83.5 ± 9.8 points (Table 4), and the correct answer rate of the tongue color discrimination question was 81.3% [(a) *n* = 16 (15%); (b) *n* = 0; (c) *n* = 0; (d) *n* = 4 (3.7%); and (e) *n* = 87 (81.3%)].

**Table 4:** Comparison of test taker's background factors (Study 2).

		All	Correct answer group	Incorrect answer group	*p* value
Medical professionals	Number of test takers^a^	107	87	20	
	Sex (male/female)	59/48	48/39	11/9	0.89*
	Age (20–29/30–39/40–49/50–59/60<)	1/29/30/30/17	1/23/24/26/13	0/6/6/4/4	0.88*
	Attendance of common Kampo lectures (yes/no)	47/60	38/49	9/11	0.91*
	Kampo specialist qualification (yes/no)^b^^,^^c^	31/51	26/40	5/11	0.55*
	Years of experience prescribing Kampo products (<3/3–<5/6)^b^^,^^c^	11/18/53	11/13/43	0/5/10	0.17*
	Number of Kampo prescriptions (<5/5–20/21<)^b^	8/32/43	4/26/37	4/6/6	0.06*
	Total test score (mean ± SD)	83.5 ± 9.8	85.3 ± 8.4	75.8 ± 11.8	<0.01**
Students	Number of test takers	56	46	10	
	Sex (male/female)	27/29	24/22	3/7	0.2*
	Age (<19/20–24/25<)	10/36/10	8/31/7	2/5/3	0.49*
	Attendance of common Kampo lectures (yes/no)	25/31	21/25	4/6	0.75*
	Total test score (mean ± SD)	72.2 ± 17.5	73.4 ± 18.2	67.0 ± 13.4	0.30**

^a^ Test takers consist of 83 medical doctors, 17 pharmacists, 5 practitioners in acupuncture and moxibustion, and 2 others.

^b^ The analysis was performed using the data of 83 medical doctors.

^c^ One test taker did not answer the question.

* Chi-square test; **t-test.

Among the students, the average score of the Kampo common test was 72.2 ± 17.5 points, and the correct answer rate for the tongue color identification question was 82.1% [(a) *n* = 10 (14.8%); (b) *n* = 0; (c) *n* = 0; (d) *n* = 0; and (e) *n* = 46 (82.1%)].

#### 3.2.2. Difficulty level and DI

For medical professionals, the correct answer rate for the question was 81.3%, and the difficulty level was moderate. The DI was 0.35, and its ability was considered good. The correct answer rate for students was 82.1%, and the difficulty level was moderate. The DI was 0.13, and its ability was poor.

#### 3.2.3. Analysis of factors related to the correct answer rate

Table 4 shows the background factors of the correct and incorrect answer groups. In the analysis of medical professionals, the total test score was significantly higher in the correct answer group than in the incorrect answer group (85.3 ± 8.4 points vs. 75.8 ± 11.8 points, *p* < 0.01). There were no significant differences in the following items between groups: sex (*p* = 0.89), age (*p* = 0.88), attendance of lectures (*p* = 0.91), KS qualification (*p* = 0.55), years of experience in prescribing Kampo products (*p* = 0.17), and the number of Kampo prescriptions (*p* = 0.06).

In the analysis of students, there was no difference in the following items between groups: sex (*p* = 0.2), age (*p* = 0.49), attendance of lectures (*p* = 0.75), and overall test score (*p* = 0.30).

## 4. Discussion

### 4.1. Current state of research on the development of equipment for tongue diagnosis

In recent years, many studies on tongue diagnosis, such as tongue diagnostic equipment (20–22), automatic diagnostic systems using artificial intelligence (23–25), and remote diagnosis using smartphones (26), have been conducted. The diagnostic ability of computers is rapidly improving, and the feasibility of standardization of tongue diagnosis using machines has been sought (27). However, only a few research studies have been conducted on educational methods and equipment to improve the ability and skill of human tongue diagnosis (28–30).

### 4.2. Practicality and reliability of the electronic evaluation system

We developed a Kampo e-learning system utilizing Moodle (https://moodle.org), which is an open-source e-learning platform, and reported its educational usefulness (10, 17, 31, 32). In this study, we evaluated tongue diagnosis ability using a Kampo e-learning/e-assessment system based on a standardized tongue image database and verified its practicality and reliability. The advantages of the electrical assessment test (i.e., online test) are that (i) the evaluation method is fair and impartial to the examinee, (ii) the diagnosis ability can be evaluated during a short interval, (iii) it is easy for both test takers and test managers to manage and utilize the grade data, (iv) it is easy to evaluate and control the test quality, (v) the test can be conducted at any time and place, and (vi) the test can be handled by a large number of test takers. System failures occur due to various causes, including operational errors, equipment failures, overloads beyond expectations, rapid increases in usage, software errors, external attacks *via* the Internet, and malware. However, we conducted the online test on approximately 160 examinees nationwide and confirmed its practicality without any trouble.

Moreover, the reliability of this test could be confirmed by analyzing the test taker's grade distribution and the difficulty of the test from various angles. The correct answer for this test was based on data determined by most diagnoses of multiple specialists (i.e., the data with the highest diagnostic concordance rate among diagnosticians). By repeating this method, the reliability and objectivity of the test can be further improved. However, the accuracy and reliability of the tongue image database depend on the diagnostic accuracy of the information providers. Qi et al. developed a method to identify a group of experts with high diagnostic accuracy and reliability by optimizing the diagnostic match score within and between diagnosticians. They stated that the comprehensive consideration of both the internal and external consistencies of each expert's diagnostic results contributes to improving the accuracy of the expert opinion-based tongue image database (33).

### 4.3. Reliability of tongue diagnosis

In a review article on the reliability of traditional East Asian medicine diagnoses, O’Brien et al. stated that studies on the reliability of tongue diagnosis and other diagnostic data collected in Chinese medicine examinations suggest considerable variability (34). Kim et al. indicated that the inadequate operational definitions of tongue characteristics and tongue inspection regions are the reason for the low levels of inter- and intrapractitioner agreement of tongue diagnosis (35). Wang et al. evaluated the reliability of doctors’ tongue diagnoses using tongue images taken with smartphones and reported that the intrarater reliability was good to very good (*κ* range, 0.7–1.0), except for the color of the tongue body (*κ *= 0.22) and slippery tongue fur (*κ *= 0.1). They also stated the interrater reliability for tongue coating was moderate (Gwet AC2 range, 0.49–0.55), whereas that for color and other features of the tongue body was fair (Gwet AC2 = 0.34) (36).

Recently, computer-based tongue image analysis techniques have improved, making it possible to quantify and identify tongue color, morphology, and features (20–25, 37–39). Analytical methods based on machine learning and deep learning have become more sophisticated, and the accuracy of machine diagnosis of tongue findings is rapidly improving. Lo et al. compared the match rate of tongue diagnosis between machines and humans and found that the intra-agreement of the automatic tongue diagnosis system was significantly higher than that of the traditional Chinese medicine practitioner (*κ* coefficient, 0.93 ± 0.06 vs. 0.64 ± 0.13) (40).

In our study, although the average percentage of the diagnostic consistency of the five KS-D participants was approximately 66%, the interrater reliability was fair for dryness and wetness of the tongue body and coating (Gwet AC1, 0.27, 0.38) and moderate for both morphological features (Gwet AC1 range, 0.45–0.53) and chromatic features (Gwet AC1 range, 0.42–0.50). If the findings of tongue imaging are complicated to diagnose, the interrater diagnostic match rate will decrease. Therefore, although it is impossible to make a uniform comparison with other research reports, it is suggested that the standard level of interrater diagnostic reliability by the naked eye of Kampo experts may be moderate. It should be noted that the moderate rating in this study is not so bad compared with similar studies in the field of Western medicine (41).

In addition, understanding the inference process of tongue diagnosis is important for developing educational equipment. Anastasi et al. verbalized the cognitive thinking process during tongue diagnosis using the think-aloud method and analyzed the differences between the diagnostic reasoning processes of novices and experts (42). They elucidated that experts use systematic reasoning patterns to determine diagnoses associated with the evaluation of tongues and indicated that these processes are congruent with those observed in Western medicine, whereby clinician reasoning involves a combination of analytical reasoning of domain knowledge and the use of exemplar patterns (42). To evaluate the ability of advanced tongue diagnosis, it is necessary to evaluate the interpretation of tongue characteristics and the comprehensive diagnostic ability, including the clinical reasoning process.

### 4.4. Discrimination ability of tongue morphology and wetness

Tongue size is often judged based on the width of the corners of the mouth, but there are no diagnostic criteria, and it is subjectively evaluated. Gwet AC1 for tongue size was 0.47, with moderate interrater reliability. Gwet AC1 for tooth marks was 0.53, showing the highest interrater reliability. A tooth mark is a pressure mark by teeth on the margin of the tongue. It occurs when the tongue retains fluid and is pressed firmly against the teeth. In Kampo medicine, it means a lack of energy or water stagnation. There are no diagnostic criteria, and the severity is subjectively assessed by the degree and number of tooth marks. Strong depressions are easily recognized because the mucosal surface turns dark red (43). Gwet AC1 for cracks was 0.46, and the interrater reliability was rated as moderate. Cracks are grooves or fissures of varying depth, shape, and number on the surface of the tongue. Fissured tongue is a normal variant seen in up to 20%–30% of the population (44). In Kampo medicine, it means a state in which the surface of the tongue is not nourished due to a lack of energy and a state in which water is deficient. Several classifications have been proposed (45, 46), and their use is expected to improve diagnostic accuracy. Gwet AC1 values for dryness and wetness of the tongue body and tongue coating were both low, 0.38 and 0.27, respectively, suggesting that evaluation of the degree of wetness is difficult. There is a high correlation between the degree of glossiness and the amount of water on the tongue surface (20), and when the tongue surface is sufficiently moistened with saliva, the glossiness of the tongue surface increases. Gwet AC1 for tongue coating thickness was 0.45, and the interrater reliability was moderate. A normal tongue is evenly covered with a very thin coating through which the tongue body can be observed. Shimizu et al. proposed a method to classify the thickness of the tongue coating according to whether the tongue papillae are visible and reported that the interobserver and intraobserver agreements were 0.66 and 0.80 for Cohen's kappa, respectively (47). Currently, in Japan, a standard method of describing tongue examination findings has been studied (48). It is necessary to create standardized diagnostic reference images for evaluating the degree of tongue morphology.

### 4.5. Discrimination ability of tongue color

In Study 2, approximately 80% of examinees correctly identified the tongue color of healthy subjects. However, most of the answerers answered incorrectly chose “pale” not “red to deep red” or “purple.” Accordingly, it is considered that the difficulty in distinguishing between light red and pale red was the main cause of the wrong answer rate, not the lack of knowledge that the tongue color of a healthy subject was light red. In Study 1, 2–5 of 15 KSs (13%–33%) selected a pale tongue in the question where the light red tongue was the answer (image 5, image 1, and image 10; Figure 7). This indicates that the visual diagnosis of light red or pale red was a relative judgment.

Traditionally, a healthy tongue has a light red body without swelling or emaciation, accompanied by a pale white thin coating. It is also mildly moist (43, 49). It is also known that tongue color varies depending on gender, age (50), and menstrual cycle (51). “Light red” and “pale,” which are considered colors of the tongue, both belong to the hue of red, but there are changes in continuity due to different lightness and saturation, and there is no clear boundary. This is the reason why color judgments have to be relative.

Typical tongue color specimen images are useful for tongue color discrimination and should be used to improve diagnosis accuracy. In Japan, five standard tongue colors have been proposed by the Tongue Diagnosis Research Group of the Ministry of Health, Labor and Welfare (8, 48). In this study, sample images were determined from the results of a majority vote of 15 KSs (Figure 7). However, in the mechanical tongue diagnosis, it is essential to quantify the typical tongue color. Wang et al. performed a statistical analysis of tongue color distribution characteristics based on more than 9,000 tongue images. They described tongue color mathematically using a color space (CIE chromaticity diagram), which is expressed as a coordinate axis in space. For example, light red is represented by 227, 150, and 147 in the red green blue color space and 69.4695, 28.4947, and 13.3940 in the lab color space (52).

Furthermore, it should be noted that color is not a physical quantity such as length or weight but a sense of vision, and there are individual differences in the sense of colors or color discrimination ability. The prevalence of congenital color blindness was 8% in men and 0.5% in women (53). Moreover, the color discrimination ability declined with age (54). Oji et al. investigated the tongue color discrimination ability of Kampo medicine practitioners and found that color discrimination declines with age but is maintained with more than 10 years of clinical experience in tongue diagnosis (55). This result shows that experience, knowledge, and training improve the ability to discriminate tongue colors.

Thus, the following factors can cause tongue color diagnosis to vary: (i) characteristics of the tongue to be evaluated (i.e., the color of the tongue is not uniform and complicated); (ii) evaluation method (i.e., the evaluation method such as the criteria and location for judging the tongue color is not determined); (iii) human characteristics (i.e., humans judge colors sensuously by color vision, and humans cannot discriminate colors in detail); and (iv) the essence of tongue examination (i.e., there is no truly correct answer). It is the collective intelligence of experience-based knowledge shared among doctors.

### 4.6. Utilization for the standardization of tongue diagnosis

The features of the system described in this study are as follows: (i) this system helps evaluate the tongue diagnosis ability of individual examinees based on information in the database, which is the collective knowledge of specialists. In this study, we demonstrated the practicality of this system as an evaluation device and its usefulness as an educational one. (ii) This system has the ability to aggregate the diagnostic skills of individual experts in a database and convert them into a collective intelligence of a group of experts. Figure 7 shows a concrete example of such results. By repeating steps (i) and (ii), the accuracy of the database can be further improved. Moreover, steps (i) and (ii) are necessary for standardizing tongue diagnosis. The standardization of tongue diagnosis is a work process that aggregates and generalizes the knowledge and skills of individual specialists as the collective intelligence of a group of specialists. Education is the work of disseminating and generalizing collective intelligence and is included in standardization. The level of skills possessed by a group of professionals will be enhanced by increasing the sharing of standardized knowledge in the professional community. This system is considered indispensable for advancing the standardization of tongue diagnosis.

## 5. Conclusion

We created a tongue diagnosis e-learning/e-assessment system based on a standardized tongue image database, conducted tongue diagnosis tests using this system among KSs, medical professionals, and students, and verified its practicality as an evaluation tool for tongue diagnosis ability. Analyzing the answer data of 15 KSs revealed that the reliability of tongue diagnosis among diagnosticians was moderate. This system can easily and objectively evaluate the tongue diagnosis ability of the examinee at any time and place and has high practicality. Utilizing this system for tongue diagnosis education and lifelong learning is expected to contribute to improving learners’ tongue diagnosis ability and the standardization of Kampo medicine.

## Data Availability

The original contributions presented in the study are included in the article/Supplementary Material, further inquiries can be directed to the corresponding author.
